# Steroid Metabolism in Children and Adolescents With Obesity and Insulin Resistance: Altered SRD5A and 20α/20βHSD Activity

**DOI:** 10.3389/fendo.2021.759971

**Published:** 2021-10-26

**Authors:** Marta Sumińska, Rafał Podgórski, Piotr Fichna, Marta Fichna

**Affiliations:** ^1^ Department of Pediatric Diabetes and Obesity, Institute of Pediatrics, Poznan University of Medical Sciences, Poznan, Poland; ^2^ Centre for Innovative Research in Medical and Natural Sciences, University of Rzeszow, Rzeszow, Poland; ^3^ Department of Biochemistry, Institute of Medical Sciences, Collegium of Medical Sciences, University of Rzeszow, Rzeszow, Poland; ^4^ Department of Endocrinology, Metabolism and Internal Medicine, Poznan University of Medical Sciences, Poznan, Poland

**Keywords:** children, adolescents, obesity, insulin resistance, 20α-hydroxysteroid dehydrogenase, 20β-hydroxysteroid dehydrogenase, 5α-reductase, urinary steroid metabolites

## Abstract

Alterations in glucocorticoid metabolism may contribute to the development of obesity and insulin resistance (IR). Obesity in turn affects the androgen balance. The peripheral metabolism of steroids is equally an important determinant of their bioavailability and activity. The aim of this study was to evaluate steroid metabolism in obese children and to define which enzyme alterations are associated with IR. Clinical characteristics and anthropometric measurements were determined in 122 obese children and adolescents (72 girls, 50 boys) aged 8 – 18 years. 26 of them (21.3%) were diagnosed with IR (13 boys, 13 girls). Routine laboratory tests were performed and 24h urinary steroid excretion profiles were analyzed by gas chromatography/mass spectrometry. Positive relationship between 5α-reductase (SRD5A) activity and IR was found. According to the androsterone to etiocholanolone (An/Et) ratio the activity of SRD5A was significantly increased in obese children with IR, but the difference remained insignificant once the 5α-dihydrotestosterone to testosterone (5αDHT/T) ratio was considered. Furthermore, this relationship persisted in boys but was not observed in girls. The activity of 20α-hydroxysteroid dehydrogenase (20αHSD) and 20β-hydroxysteroid dehydrogenase (20βHSD) was reduced only in obese girls with IR. Conclude, in the context of obese children and adolescents with IR, we surmise that increased SRD5A represents a compensatory mechanism to reduce local glucocorticoid availability. This phenomenon is probably different in the liver (restriction) and in the adipose tissue (expected increase in activity). We show significant changes in 20αHSD and 20βHSD activity in obese girls with IR, but it is difficult to clearly determine whether the activity of these enzymes is an indicator of the function in their ovaries or adrenal glands.

## Introduction

In 1974, experts from the World Health Organization (WHO) placed obesity on the infamous list of civilization diseases. Unfortunately, since then, the problem of excess body mass is still increasing among populations worldwide, including children. According to the data from 2016 the number of obese children (aged > 5 years) and adolescents has increased since 1974 from 11 to 124 million nowadays, and a further 213 million are overweight (1). In Poland, the problem of excess body mass, according to various studies (HBSC 2014/2018, COSI, PITNUTS 2016), affects about 28% of young children, 30% of early school-age children and on average some 20% of adolescents up to 15 years of age.

It is well established that steroid hormones play an important role in determining body fat distribution (2–5). Many studies have also proved that obesity is associated with abnormalities in the hypothalamic-pituitary-adrenal (HPA) axis including alterations in diurnal cortisol rhythm and enhanced susceptibility of the HPA axis for activation (4, 6–9). All of the above-mentioned mechanisms may escalate tissue exposure to glucocorticoids. However, circulating cortisol concentrations in obese individuals are reported to be within the reference range (10–14). Adipose tissue dysfunction characterized by increased visceral fat accumulation, larger adipocyte size and more abundant macrophage infiltration of the omental fat is associated with insulin-resistant obesity (15). Klöting et al. demonstrated increased macrophage infiltration into omental compared with subcutaneous adipose tissue, which may provide explanation why many individuals with subcutaneous obesity seem to be spared from insulin resistance (IR) and other adverse metabolic effects (15, 16). The molecular mechanisms determining glucocorticoids impact on diverse adipose tissue subsets are poorly understood. Bidirectional interaction between endogenous glucocorticoids and fat tissue in the coexistence of IR can be explained by elevated GLUT4 expression in the human visceral adipose tissue. Lundgren et al. showed that omental adipocytes, an observation display approximately 2-fold higher glucose uptake rate compared with subcutaneous adipocytes which was additionally supported by the increased number of GLUT4 receptors (17). Furthermore, glucocorticoids exert a marked suppressive action on glucose uptake and the expression of insulin signaling proteins in omental but not in subcutaneous adipocytes (5, 17). There is no doubt that steroids and obesity are linked by several, not fully explained, ways.

So called adrenal androgens, are in fact precursors of the more potent androgens, released from the adrenal cortex in a parallel way to glucocorticoids. Both, adrenal androgens and glucocorticoids are substrates for the same enzymes, however, the effect of their action may be opposite – androgen activation, but loss of glucocorticoid power.

11β-hydroxysteroid dehydrogenases (11βHSD) are enzymes implicated in steroid hormone balance. 11β-hydroxysteroid dehydrogenase type 1 (11βHSD1) predominantly acts as a reductase by recovering cortisol from inactive cortisone and has been localized in the liver, adipose tissue and central nervous system. Tissue-specific expression of 11βHSD1 can enhance local cellular availability of glucocorticoids. 11β-hydroxysteroid dehydrogenase type 2 (11βHSD2) is an enzyme expressed in the epithelial tissues such as kidney, colon, salivary and sweat glands. Its action leads to the oxidation of cortisol to cortisone and prevents excess activation of the mineralocorticoid receptors by cortisol (18, 19).

5α-reductase (SRD5A) is an enzyme widely known for converting testosterone into 5α-dihydrotestosterone (5αDHT), however, it can also exert an effect on other steroids, including several androgen precursors and cortisol (20). SRD5A presents under three isoforms. 5α-reductase type 1 (SRD5A1) is responsible for conversion of androstenedione to androsterone. It occurs mainly in the skin and to a lower extent in the prostate gland. 5α-reductase type 2 (SRD5A2) is involved in the activation of testosterone to the most potent 5αDHT as mentioned above. This enzyme is expressed in the testes, prostate and genital skin. Additionally, both isoenzymes, type 1 and type 2 are found in the liver. The role of 5α-reductase type 3 (SRD5A3) remains unclear, although it appears ubiquitously expressed in human tissues (21). 5α-reductase often co-occurs in duet with 5β-reductase: both enzymes represent a convergence in evolution: they share similar biological functions, but do not have a common ancestor (22).

The 3β-hydroxysteroid dehydrogenase (3βHSD) is a key enzyme in the synthesis of all active steroid hormones, such as glucocorticoids, mineralocorticoids, progesterone, androgens and estrogens (23, 24). No less important function is the hepatic degradation of the androstenone (25). 3βHSD controls critical steroid hormone-related reactions in the adrenal cortex, gonads, placenta, liver, and other peripheral target tissues (24, 26, 27).

Details of the activities of 20α-hydroxysteroid dehydrogenase (20αHSD) and 20β-hydroxysteroid dehydrogenase (20βHSD) remain unclear. The cortols are metabolites of tetrahydrocortisol (THF) and α-tetrahydrocortisol (αTHF) after degradation by 20αHSD or 20βHSD, while the cortolones are metabolites of tetrahydrocortisone (THE) after the action of the same enzymes respectively. The multispecificity of enzymes, especially 20βHSD, appears to imply different physiological roles in various species and alternative effects on steroids metabolism (28–32).

11β-hydroxylase is a steroid enzyme found in the zona glomerulosa and zona fasciculata of the adrenal cortex. There are two isozymes encoded by the CYP11B1 and CYP11B2 genes on human chromosome 8q. The first is involved in the biosynthesis of adrenal corticosteroids, mainly for the conversion of 11-deoxycortisol into cortisol. It is regulated by ACTH (33). Dysfunction of this enzyme is associated with congenital adrenal hyperplasia (34–36). The second isoform is expressed at low levels in the normal adrenal zona glomerulosa, but at higher levels in aldosterone-secreting tumors (33).

17β-hydroxysteroid dehydrogenase (17βHSD) controls the last step in the formation of all androgens and estrogens. It is involved in both: the activation and inactivation of this hormones. Fourteen isoforms of this enzymes have been identified, encoded by HSD17B1 to HSD17B14 genes. Human adipose tissue is capable of active androgen synthesis catalyzed by selected 17βHSD isoforms (37, 38). The intraabdominal adipose tissue may be substantially androgenic, increasingly so with growing obesity, particularly central obesity.

The peripheral metabolism of steroids is a considerable determinant of their availability and may be responsible for altered activity of those hormones. The aim of this study was to evaluate steroid metabolism in obese children and to investigate which enzymatic alterations can be associated with IR in this population.

## Research Design and Methods

The study comprised 122 patients (70 girls, 52 boys) aged between 5 and 18 years (mean 12.0 +/- 3.5) suffering from childhood obesity (**Table 1**). Obesity was defined as BMI values above the 97th percentile of the BMI reference curve based on percentile scales developed for the population of Polish children and adolescents. The group was further stratified according to the presence or absence of IR. Individuals with obesity secondary to an underlying endocrine disorder or genetic syndromes, as well as those under current medication were excluded from the study. All patients underwent routine clinical assessment including general physical examination, basic anthropometric measurements and pubertal stage evaluation based upon Tanner scale. The examination was followed by blood sampling after overnight fast and 24h urine collection according to standard protocol. The study was conducted in line with the Declaration of Helsinki and approved by the Bioethical Committee at Poznan University of Medical Sciences. Informed consent was obtained from the participants aged at least 16 years old and, in case of minors, from their legal representatives.

**Table 1:** Clinical phenotype of the non-insulin resistant (non-IR) and insulin resistant (IR) groups.

	Non – IR (n = 96)		IR (n = 26)		*p* value
Sex (male/female)	39/57		13/13		0.464
Tanner 1/2/3/4/5 (%)	32/15/11/13/29		30/17/17/17/17		0.620
	Mean + SD	Median (IQR)	Mean + SD	Median (IQR)	
Age (years)	12.2 ± 3.6	13 (9-15)	11.4 ± 3.4	12 (9-14)	0.242
Duration of obesity (years)	6.6 ± 3.6	5 (4-9)	7.6 ± 3.5	7 (5-9)	0.122
BMI (kg/m^2^)	28.6 ± 5.4	28.0 (25.0-31.2)	33.4 ± 7.4	32.1 (27.1-37.4)	**0.002**
Z-score BMI	2.1 ± 0.4	2.1 (1.9-2.4)	2.5 ± 0.4	2.5 (2.2-2.7)	**<0.001**
WHR	1.0 ± 0.8	0.9 (0.8-0.9)	0.9 ± 0.0	0.9 (0.9-1.0)	0.285
CHOL (mg/dl)	175.5 ± 31.4	169.0 (157.0-196.0)	179.4 ± 31.3	176.0 (157.0-204.0)	0.719
LDL (mg/dl)	107.2 ± 27.5	102.5 (90.7-120.2)	108.1 ± 24.8	104.0 (94.0-127.0)	0.977
HDL (mg/dl)	45.8 ± 8.7	45.0 (40.0-51.2)	43.6 ± 10.4	43.0 (35.0-52.0)	0.331
TG (mg/dl)	109.7 ± 57.3	96.0 (72.5-128.0)	138.8 ± 83.4	107.0 (87.0-171.0)	0.117
Fasting glucose (mg/dl)	87.7 ± 9.1	87.0 (83.0-91.0)	92.5 ± 6.7	93.0 (88.5-97.5)	**0.005**
Fasting insulin (mU/ml)	11.2 ± 4.3	10.0 (7.8-13.9)	25.8 ± 6.7	23.9 (22.1-27.6)	**<0.001**

Statistically significant differences were bolded.

### Biochemical Analyses

All biochemical measurements were performed in laboratory of the university reference hospital. Glucose, insulin levels and lipids profile were assessed after overnight fast (**Table 1**). Standard colorimetric method was used for determination of plasma glucose level (Clinical Chemistry Analyzer AU680, Beckman Coulter). Serum insulin was measured by the chemiluminescence method (Alinity i, Abbott). Insulin resistance was evaluated based upon fasting plasma glucose (FPG) and insulin (FPI) with homeostasis model assessment for IR index (HOMA-IR) (39, 40). For our study IR was defined as HOMA-IR > 97th percentile together with concomitant FPI > 15 μU/mL (41–44).

### Quantification of Urinary Steroid Metabolites

24-hour urine collections were performed at home to avoid extra stress connected with hospitalization. To ensure compliance children and parents were primed in the collection procedure and also received written instructions.

Samples were stored at -20˚C until analyzed by the in-house adapted gas chromatography-mass spectrometry (GC-MS) method as previously described (45–47). All measurements were performed in the same laboratory in the Centre for Innovative Research in Medical and Natural Sciences at the University of Rzeszow, Poland. A list of measured urinary steroid metabolites is presented in **Table 2**.

**Table 2:** List of steroid compounds measured in urines.

Trivial name	Abbreviation	Systematic name	M	RT	QIon	Ref. Ion
Cortisol	F	4-pregnen-11β, 17, 21-triol-3, 20-dione	362.5	29.2	606	488
Cortisone	E	4-pregnen-17, 21-diol-3, 11, 20-trione	360.5	27.2	532	515
Tetrahydrocortisol	THF	5β-pregnan-3α, 11β, 17, 21-tetrol-20-one	366.5	23.3	653	472
5α-Tetrahydrocortisol	5αTHF	5α-pregnan-3α, 11β, 17, 21-tetrol-20-one	366.5	23.6	653	551
Tetrahydrocortisone	THE	5β-pregnan-3α, 17, 21-triol-11, 20-dione	364.5	21.9	579	488
Androsterone	An	5α-androstan-3α-ol-17-one	290.4	13.5	270	360
Etiocholanolone	Et	5β-androstan-3α-ol-17-one	290.4	13.7	270	360
5α-Dihydrotestosterone	5αDHT	5α-androstan-17β-ol-3-one	290.4	15.2	391	360
Testosterone	Testosterone	4-androsten-17β-ol-3-one	288.4	15.6	389	268
Androstenetriol	AET	5-androsten-3β, 16α, 17β-triol	306.4	19.3	432	522
16α-Hydroxy-DHA	16α-OH-DHA	5-androsten-3β, 16α-diol-17-one	304.4	17.1	266	446
11ß-Hydroxyandrosterone	11β-OH-An	5α-androstan-3α, 11β-diol-17-one	306.4	16.4	268	448
11ß-Hydroxyetiocholanolone	11β-OH-Et	5β-androstan-3α, 11β-diol-17-one	306.4	16.7	268	448
α-Cortol	αC	5β-pregnan-3α, 11β, 17, 20α, 21-pentol	368.5	25.7	343	563
ß-Cortol	ßC	5β-pregnan-3α, 11β, 17, 20β, 21-pentol	368.5	24.7	343	551
α-Cortolone	αCl	5β-pregnan-3α, 17, 20α, 21-tetrol-11-one	366.5	24.0	449	523
ß-Cortolone	ßCl	5β-pregnan-3α, 17, 20β, 21-tetrol-11-one	366.5	24.8	449	253
Stigmasterol (IS)	SS	5, 22-cholestadien-24β-ethyl-3β-ol	412.7	28.3	394	353
Cholesterol N-butyrate (IS)	CB	5-cholesten-3β-ol n-butyrate	456.8	31.9	368	360
Medroxyprogesteron (IS)	MP	4-pregnen-6α-methyl-17-ol-3, 20-dione	344.5	21.8	443	–

M, molar mass [g/mol]; RT, Retention time [min]; QIon, quantifier ion [m/z]; Ref. Ion, Reference Ion [m/z]. OH, hydroxy; DH, dihydro; TH, tetrahydro.

All steroids standards including medroxyprogesterone, cholesteryl butyrate and stigmasterol were obtained from Steraloids (Newport RI, USA), the Sep-Pak C18 column from Waters (Milford, MA, USA), the Lipidex 5000 from Perkin Elmer (Waltham MA, USA), while β-glucuronidase/arylsulfatase liquid enzyme, the powdered sulfatase type H-1 enzyme from Helix pomatia, the derivative agents methoxyamine hydrochloride and trimethylsilylimidazole (TMSI), pyridine, sodium acetate and acetic acid were provided by Sigma - Aldrich (Darmstadt, Germany).

In brief, the method comprises a pre-extraction of a urine sample on a Sep-Pak C18 cartridge with the recovery standard medroxyprogesterone, enzymatic hydrolysis with sulfatase and β-glucuronidase/arylsulfatase, and subsequent extraction of the free steroids again on a Sep-Pak C18 column, derivatization with methoxyamine hydro-chloride 2% in pyridine at 60˚C for 3 hours and next with TMSI at 100˚C for 16 hours after adding the two internal standards Stigmasterol and Cholesteryl butyrate. Eventually, the sample is purified on a Lipidex 5000 column.

GC-MS was performed with a Shimadzu 2010 Plus gas chromatograph (Kyoto, Japan) interfaced with a single-quadrupole Shimadzu QP-2010 Ultra mass spectrometer. Data were acquired at 70 eV, the ion source temperature was 230°C and the interface temperature was 250°C. The samples (2 µl) were injected using Shimadzu AOC-20i au-to-injector in spitless mode at 260°C and separated through a ZB-1ms (15 m × 0.25 mm I.D., 0.25 µm film thickness - Pheanomenex, Torrance, USA) cross-linked dimethylpolysiloxane capillary column.

The injection was performed at 50°C which was held for 3 minutes, raised to 210°C at 30°C/min, next to 265°C at 2°C/min and finally increased to 320°C using a 20°C/min ramping program over a 48-min period. Helium was used as the carrier gas in linear velocity flow control mode. The flow of gas through the column was 1.2 ml/min. The eluting steroids were detected by selected ion monitoring (SIM). The specific ions used for the determination of each steroid are listed in **Table 2**. The instrument was calibrated by analyzing standard mixtures containing known amounts of the reference steroids and internal standards. The area of the obtained peaks was measured in SIM mode and a six-level calibration curve was set for each analyte. Recoveries were checked with medroxyprogesterone and corrections were made for the losses occurring during sample preparation. **Table 1** from **Supplementary Material** provides method-validation information for all quantified steroids. For all measured steroids, specific peaks in the chromato-grams had to be at least three times higher as the noise above the baseline to be count-ed as valid measurements (signal-to-noise-ratio 3). Urine samples of the same healthy volunteer were measured in all series and the results were compared with the standard values derived from 10 former measurements of this volunteer. For quality control, quantitative results had to be within ± 30% of the individual reference intervals.

### Proportion of Urinary Corticosteroid Metabolites

In order to evaluate enzyme activity, we applied proportions of the excreted steroid metabolites (32, 48, 49). The ratios of cortisol (F) to cortisone (E) as well as tetrahydrometabolites of cortisol [THF+5αTHF] to this of cortisone [THE] reflect 11βHSD1 activity. The activities of 5α- and 5β-reductases, located mainly in the liver, can be inferred from the ratio of 5α-dihydrotestosterone (5αDHT) to testosterone (T) and androsterone (An) to etiocholanolone (Et). By analogy, we hypothesize that the 11ß-hydroxyandrosterone (11β-OH-An) to 11ß-hydroxyetiocholanolone (11β-OH-Et) ratio will reflect the 5α-reductase activity and – indirectly – the androgen metabolism after their second crossing through the adrenal cortex. Only 5α-reductase can be evaluated with the androgen ratios, while 5β-reductase predominates for cortisol and cortisone in cooperation with 3αHSD. The activity of the 5β-reductase+3αHSD complex was assessed using the ratios [α-cortol (αC)+β-cortol (βC)+THF]/F and [α-cortolone (αCl)+β-cortolone (βCl)+THE]/E. The activity of 20αHSD was assessed by the ratio of [αC+αCl]/[THF+5αTHF+THE] and the activity of 20βHSD by the [βC+βCl]/[THF+5αTHF+THE] ratio. The proportions of 11β-OH-An/An and 11β-OH-Et/Et were used to determine the activity of 11β-hydroxylase. The last enzyme we evaluated was 17βHSD, the activities of which we assessed using a ratio of androstenetriol (AET) to 16α-hydroxydehydro-androsterone (16α-OH-DHA).

### Statistical Analyses

The ratios of the 24h urinary excretion of steroid metabolites were compared using the Statistica 13.3 (StatSoft Inc, Tulsa, OK, USA). Most of the variables did not follow normal distribution hence Mann-Whitney U test was used for comparisons between patients with and without IR. The level of statistical significance was accepted as p-value < 0.05.

## Results

Of 122 obese children and adolescents 26 (21.3%) were diagnosed with IR (13 boys, 13 girls). Clinical phenotype of non-IR and IR participants is displayed in **Table 1**. The mean age, duration of obesity and WHR were not significantly different between patients with and without IR. The BMI and Z-score BMI were significantly higher in the IR group than in the non-IR group. At the biochemical level, patients with IR versus those without IR presented similar values in lipid profile but significantly higher mean values of fasting glucose and insulin level. No significant differences were found in steroid metabolites excretion between obese subjects with and without IR (**Table 2** from **Supplementary Material**).

In order to assess enzyme activities, typical metabolite ratios were calculated and compared between IR and non-IR cohorts of obese children (**Table 3**). Furthermore, gender-stratified analyses were also performed, comprising 70 girls and 52 boys divided by their insulin sensitivity status (**Tables 4** and **5**). A positive association between SRD5A activity and IR was noticed in our study. The activity of 5α-reductase, measured regardless of gender, was increased in children with IR when based upon the An/Et ratio, but statistical significance was lost when 5αDHT/T and 11β-OH-An/11β-OH-Et ratio were considered. Similar relationship persisted in the group of obese boys, whereas we could not confirm it in obese girls. The activity of 20αHSD, measured by the [αC+αCl]/[THF+5αTHF+THE] ratio and the activity of 20βHSD, measured by the [βC+βCl]/[THF+5αTHF+THE] ratio were both reduced only in the group of girls with IR when compared to non-IR girls, whereas the same ratios remained similar in obese boys with and without IR. We failed to confirm statistically significant differences in the activity of 11βHSD1, 5β-reductase/3αHSD complex, 11β-hydroxylase and 17αHSD between IR and non-IR children (data not shown), therefore no further analyses of these enzymes were performed in our cohort.

**Table 3:** Ratios of steroid metabolites (indicative of enzyme activity): comparison between obese children and adolescents with and without insulin resistance (IR).

	Non – IR (n = 96)		IR (n = 26)		*p* value
	Mean + SD	Median (IQR)	Mean + SD	Median (IQR)	
**SRD5A activity**					
Androsterone/Etiocholanolone	1.84 ± 0.86	1.67 (0.37-4.75)	2.50 ± 1.11	2.39 (0.81-4.93)	**0.023**
5α-DHT/Testosterone	0.98 ± 1.01	0.63 (0.32-1.22)	0.96 ± 0.78	0.84 (0.42-1.17)	0.600
11β-OH-An/11β-OH-Et	5.65 ± 5.35	3.69 (0.48-24.74)	8.88 ± 13.45	4.55 (0.90-51.74)	0.496
**20α-HSD activity**					
[αC+αCl]/[THF+αTHF+THE]	0.35 ± 0.12	0.32 (0.13-0.76)	0.35 ± 0.19	0.30 (0.08-1.02)	0.502
**20β-HSD activity**					
[βC+βCl]/[THF+αTHF+THE]	0.19 ± 0.10	0.17 (0.02-0.68)	0.17 ± 0.09	0.13 (0.08-0.49)	0.126

Statistically significant differences were bolded.

**Table 4:** Ratios of steroid metabolites (indicative of enzyme activity): comparison between obese boys with and without insulin resistance (IR).

	Non – IR (n = 39)		IR (n = 13)		*p* value
	Mean + SD	Median (IQR)	Mean + SD	Median (IQR)	
Age (years)	11.7 ± 3.4	13 (9-14)	11.7 ± 2.7	12 (11-14)	0.818
BMI (kg/m^2^)	29.8 ± 6.1	28.4 (26.0-32.1)	32.8 ± 7.2	32.0 (28.5-36.3)	**0.028**
Z-score BMI	2.2 ± 0.6	2.2 (1.9-2.4)	2.4 ± 0.4	2.3 (2.2-2.6)	0.134
**SRD5A activity**					
Androsterone/Etiocholanolone	1.90 ± 0.97	1.73 (0.67-4.75)	2.76 ± 0.97	3.11 (1.46-4.17)	**0.035**
5α-DHT/Testosterone	0.93 ± 0.88	0.55 (0.31-1.38)	0.82 ± 0.51	0.84 (0.34-1.21)	0.867
11β-OH-An/11β-OH-Et	5.25 ± 4.45	3.81 (0.64-19.48)	7.59 ± 13.30	4.48 (0.90-49.29)	0.835
**20α-HSD activity**					
[αC+αCl]/[THF+αTHF+THE]	0.35 ± 0.14	0.32 (0.13-0.76)	0.43 ± 0.23	0.39 (0.18-1.02)	0.368
**20β-HSD activity**					
[βC+βCl]/[THF+αTHF+THE]	0.20 ± 0.11	0.17 (0.08-0.63)	0.20 ± 0.11	0.17 (0.09-0.49)	0.983

Statistically significant differences were bolded.

**Table 5:** Ratios of steroid metabolites (indicative of enzyme activity): comparison between obese girls with and without insulin resistance (IR).

	Non – IR (n = 57)		IR (n = 13)		*p* value
	Mean + SD	Median (IQR)	Mean + SD	Median (IQR)	
Age (years)	12.2 ± 3.8	13 (9-15)	11.0 ± 4.0	11 (8-14)	0.248
BMI (kg/m^2^)	29.5 ± 6.4	28.6 (25.6-33.5)	34.0 ± 7.9	33.0 (27.0-42.3)	**0.028**
Z-score BMI	1.9 ± 0.6	2.0 (1.7-2.2)	2.5 ± 0.4	2.6 (2.2-2.7)	**<0.001**
**SRD5A activity**					
Androsterone/Etiocholanolone	1.81 ± 0.78	1.64 (0.37-3.81)	2.24 ± 1.22	2.29 (0.81-4.93)	0.402
5α-DHT/Testosterone	1.01 ± 1.10	0.65 (0.34-1.21)	1.09 ± 0.98	0.84 (0.47-1.17)	0.720
11β-OH-An/11β-OH-Et	5.93 ± 5.95	3.66 (0.48-24.74)	10.06 ± 14.02	4.55 (1.59-51.74)	0.560
**20α-HSD activity**					
[αC+αCl]/[THF+αTHF+THE]	0.35 ± 0.10	0.32 (0.22-0.71)	0.28 ± 0.11	0.27 (0.08-0.51)	**0.029**
**20β-HSD activity**					
[βC+βCl]/[THF+αTHF+THE]	0.18 ± 0.10	0.17 (0.02-0.68)	0.14 ± 0.05	0.13 (0.08-0.26)	**0.021**

Statistically significant differences were bolded.

## Discussion

Childhood obesity is rapidly increasing worldwide (1). Furthermore, insulin resistance seems far more frequent in obese children and adolescents than previously considered (50, 51). However, most data about obesity, IR or other metabolic disorders in relation to steroid imbalance are still derived from the adult populations. Nonetheless, knowledge from adults cannot be directly transferred into paediatric settings, as children are not mere copies of adults. As illustrated in our study cohort, children usually present relatively short duration of obesity, its early onset, and stages of puberty. Objective assessment of IR in children remains difficult due to the developmental changes, which naturally decrease insulin sensitivity during the puberty.

As early as 1974, Savage et al. found significantly higher excretion of 17OH-corticosteroid derivatives and cortisol metabolites in obese children compared to their norm-weight coevals (52). Similar observation was described a few years later by Juricskay and Molnár, who showed increased elimination of cortisol metabolites, pregnenediol and pregnanolone together with increased excretion of adrenal androgens in obese children (53, 54). In the following years, studies were primarily focused on hyperactivity of the HPA axis in children with obesity and metabolic syndrome (55, 56). On the other hand, Vitkin et al. observed a general decrease in the excretion of gluco- and mineralocorticoid metabolites, along with an increase in androgen metabolites and enhanced activity of 17,20-lyase, 17-hydroxylase, 11βHSD1 and a decrease in the activity of 21-hydroxylase in children with obesity (57). At the same time, reports of lack of any correlation between blood cortisol values and body weight emerged - according to Knuttson, individual differences in cortisol concentration represent normal homeostasis rather than pathological phenomenon (11, 13). Another aspect i.e. the relationship between adrenal steroids or androgens and IR in children and adolescents, is even less understood. Adam et al. demonstrated that cortisol may contribute to reduced insulin sensitivity at an early age in Latino children and adolescents (3). In line, serum cortisol was moderately increased in obese children with IR, while weight reduction led to a decrease both in cortisol and IR (58). A recently published study revealed specific steroid metabolomic signature of IR in obese children (with no distinction of gender) characterized by enhanced secretion of steroids from all three adrenocortical pathways (49). This finding may suggest the hypothalamo-pituitary activation and secondary enhancement of early steps of adrenal steroidogenesis.

In our study, we characterized glucocorticoid metabolism and excretion in a cohort of young obese individuals with and without IR, also in terms of their gender. Slightly more than 1/5 of the studied group were children and adolescents with IR, which corresponds to commonly observed proportion in general practice. Considering potential differences in individual reactivity to insulin, we were rather interested in activity of particular enzymes than in differences of the concentration of excreted metabolites, which additionally may be affected by the kidney function.

Recent decades have focused on the role of 11βHSD1 in the pathogenesis of obesity and IR through locally increased cortisol levels (59). We were unable to demonstrate difference in activity of 11βHSD1 which is repeatedly mentioned in many studies in obese people with or without additional metabolic disorders (59–66). However, most of these analyses were conducted in adult populations, and compared obese with lean subjects.

As mentioned above, three isotypes of SRD5A are known. The methods used in this study do not allow to clearly distinguish between the enzymes, but we made an attempt to determine organ specificity (liver versus adrenal glands). Clinical data support the hypothesis that insulin acutely enhances ACTH effects on glucocorticoid pathways by stimulation of SRD5A activity and reduced level of the active glucocorticoid - cortisol in different organs (67). In our study we have results from the androgenic part of the steroid panel, not from glucocorticoid metabolism. Nonetheless, it is worth emphasizing that both pathways remain under control of ACTH and there is close relationship between them.

Increased excretion of 5α-reduced steroids was observed in obesity, PCOS and nonalcoholic fatty liver disease (68–70), while decreased excretion was noticed in critical illness (71). In rodents, treatment of obese Zucker rats with insulin sensitizers, decreases SRD5A1 expression in the liver (72). At the same time congenital deficiency of SRD5A1 causes intrahepatic accumulation of glucocorticoids and induces IR, hepatic steatosis and even fibrosis (73). These data further support close relationship between 5α-reductase activity, concentration of glucocorticoids in the liver and metabolic disorders.

SRD5A is a key enzyme implicated in androgen metabolism. It catalyzes the irreversible conversion of testosterone to 5αDHT (classic pathway) in androgen-dependent target tissues (74). The backdoor pathway is an alternative route to 5αDHT synthesis, which circumnavigates androstenedione and testosterone intermediates by 5α-reduction of progesterone or 17-hydroxy-progesterone into pre-androgen metabolites (75–77). In contrast to etiocholanolone, which originates almost exclusively from the classic pathway, androsterone additionally may arise from the backdoor pathway. Therefore, the An/Et ratio can be used as an indicator for the activity of the backdoor pathway (76). Previous reports demonstrated enhanced SRD5A activity in obese individuals with or without other metabolic disorders, an observation which we hereby confirm as measured by urinary An/Et ratio (48, 49, 78). Furthermore, our analysis revealed association between SRD5A activity and IR in obese subjects, but once this cohort was gender-stratified, we were only able to detect this relationship in boys. Puberty was underway in a proportion of the studied patients, which may explain the abundance of available androgenic substrates for the enzyme. Sexual dimorphism of the SRD5A activity was formerly described with increased activity in males and also in women with hyperandrogenism in the course of PCOS (32, 79–81).

20αHSD was initially described as a progesterone-metabolizing enzyme of the ovary (82). It is expressed in human endometrium and catalyzes the conversion of progesterone to its inactive form, 20α-dihydroprogesterone. Consequently, it appears to play a role during pregnancy and parturition. High 20αHSD activity has been located in the mouse adrenal cortex and liver, but the enzymatic regulation and functional significance of this tissue-restricted expression pattern remain elusive (83–85). In humans, its expression was detected in liver, brain, and to much smaller extent in adrenals and testes. Over recent years only a few reports have looked at the activity of this enzyme in humans, mostly in obese women or women with PCOS (86–89). Data on the activity of 20βHSD are even less available. In some fish species 20βHSD is a key enzyme in the production of the oocyte maturation-inducing steroid (90, 91). Vazirzadeh et al. suggest a wider metabolic role of 20βHSD than just control of synthesis of the reproduction hormones (92). Experiments on zebrafish show that 20βHSD represents a short pathway to rapidly inactivate and excrete cortisol and hence might be an important enzyme in stress response (28). No reliable data on its activity exist in humans. In our study, we show significant changes in 20αHSD and 20βHSD activity in obese girls with IR compared to those with preserved insulin sensitivity. However, it is difficult to clearly determine whether the activity of these enzymes is an indicator of the function in the ovaries or adrenal glands. In our observation, both enzymes presented diminished activity in obese girls with IR only when assessed by measurements of the ratio of cortols and cortolons to cortisol and cortisone tetrahydro-derivatives. It seems that 20αHSD and 20βHSD dis-play preferential affinity for substrates after 5α/5β-reduction and especially after 3αHSD conversion. Perhaps, insulin resistance itself contributes to enhanced activity of 3αHSD (maybe in complex action with 5β-reductase) and more substrates are available to be metabolized by 20αHSD or 20βHSD. However, there is still open question about plausible reasons of sex dimorphism of such a phenomenon.

The present results are close to the previous thesis that obesity and IR exhibit an increase in activity of SRD5A. Hence, we have added 20αHSD and 20βHSD to disease signature and provide evidence that girls with IR present reduced activity of those frequently overlooked enzymes, with a function which is still not fully understood.

Based on our study, in the context of obese children and adolescents with IR, we hypothesize that increased SRD5A activity (especially in boys) represents a compensatory mechanism to reduce local glucocorticoid availability. This phenomenon is probably different in the liver (restriction) and in adipose tissue (expected increase in activity), but requires further research, especially with regard to the various developmental stages. Increased inactivation of cortisol through enhanced SRD5A activity leads to decreased local glucocorticoid availability and their activation in the liver - in the aim to protect hepatic insulin sensitivity. The statistically irrelevant 11β-OH-An/11β-OH-Et ratio seems to exclude significant role of the local adrenal 5α-reductase activity, because these products were generated by CYP11B1/2. The decrease in activity of 20αHSD and 20βHSD characteristic for obese girls with IR remains unclear and in contrast to the results of studies in women with PCOS (32). The advancement of puberty as a factor influencing the activity of these enzymes, closely related to the reproduction, cannot be ruled out. Some of the girls were pre-pubertal or at early puberty stages, and girls who were menstruating – were evaluated in the first phase of their cycle, therefore they probably had low progesterone levels. Finally, a direct effect of insulin secretion on the activity of each of the tested enzymes cannot be excluded too.

Our observations and interpretation of the results must take into account that in children and adolescents the hormonal status is not as homogeneous and stable as in adults with obesity and IR. Conclusions from the study are mainly hypotheses, which require confirmation in further cohorts. We are aware that our study has some limitation: the small number of subjects in subgroups when the patients were divided according to gender. However, it is important to look for the mechanisms of disorders found in adults, such as obesity and IR, at their early stages, already at the developmental age. This may have an impact on the risk assessment of future complications in the adulthood. Additionally, we revealed some early gender-related differences in steroid metabolism. Indubitably, further studies are warranted to validate our findings and con-firm our assumptions.

## Data Availability Statement

The raw data supporting the conclusions of this article will be made available by the authors, without undue reservation.

## Ethics Statement

The studies involving human participants were reviewed and approved by Bioethical Committee at Poznan University of Medical Sciences. Written informed consent to participate in this study was provided by the participants’ legal guardian/next of kin.

## Author Contributions

Conceptualization – MS, PF, and MF. Patient care and data gathering – MS and PF. Laboratory GC/MS methods application – RP. Data analysis – MS, RP, and PF. Writing - original draft - MS. Writing - review and editing – RP and MF. All authors have read and agreed to the published version of the manuscript.

## Funding

This study was funded by a grant number SDUM-GB37/03/21 from Poznan University of Medical Sciences awarded to MS at the Department of Pediatric Diabetes and Obesity. The funding source had no role in the study design, data collection, data analysis, data interpretation, writing of the manuscript, or decision to publish the results.

## Conflict of Interest

The authors declare that the research was conducted in the absence of any commercial or financial relationships that could be construed as a potential conflict of interest.

## Publisher’s Note

All claims expressed in this article are solely those of the authors and do not necessarily represent those of their affiliated organizations, or those of the publisher, the editors and the reviewers. Any product that may be evaluated in this article, or claim that may be made by its manufacturer, is not guaranteed or endorsed by the publisher.
