# Supplementary material for: Steroid Metabolism in Children and Adolescents With Obesity and Insulin Resistance: Altered SRD5A and 20α/20βHSD Activity
Source: Front Endocrinol (Lausanne). 2021 Oct 26;12:759971. doi: 10.3389/fendo.2021.759971 (PMC8577858; doi:10.3389/fendo.2021.759971)
Supplement: Supplementary file 1 [file Table_1.pdf]

Supplementary Material

Table 1. Method validation information.

Trivial name	LOD	LOQ	R^2	CV1	CV2	Rec
Cortisol	367	1112	0.9987	4.1	7.7	86
Cortisone	130	394	0.9989	5.6	14.2	82
Tetrahydrocortisol	459	1391	0.9994	8.9	13.2	85
5α-Tetrahydrocortisol	407	1232	0.9996	8.2	12.1	87
Tetrahydrocortisone	699	2117	0.9998	2.5	7.7	83
Androsterone	435	1318	0.9999	3.3	7.7	87
Etiocholanolone	263	798	0.9998	3.4	5.6	86
5α-Dihydrotestosterone	562	1704	0.9994	5.0	5.8	87
Testosterone	144	436	0.9991	4.0	5.3	104
Androstenetriol	434	1316	0.9997	1.5	8.5	80
16α-Hydroxy-DHA	124	375	0.9999	3.7	11.5	82
11ß-Hydroxyandrosterone	189	573	0.9998	2.6	6.2	103
11ß-Hydroxyetiocholanolone	114	346	0.9998	4.4	6.3	95
α-Cortol	377	1142	0.9994	1.3	6.4	88
ß-Cortol	694	2103	0.9995	5.6	15.0	92
α-Cortolone	196	593	0.9991	2.2	15.5	87
ß-Cortolone	389	1179	0.9994	2.0	6.7	90

Abbreviations list: LOD: limit of detection [pg per sample]. LOQ: limits of quantitation [pg per sample], R^2: correlation coefficient of the linear calibration curve. Rec: Recovery in %. CV1: intraassay coefficient of variation [%] (n = 6). CV2: interassay coefficient of variation [%] (n = 6). CV1 and CV2 were determined for a urine volume of 1.5 mL.

Table 2. 24h urinary excretion of steroid metabolites (µg/24h) in obese children and adolescents: comparison between patients with and without insulin resistance (IR).

	Non – IR (n = 96)		IR (n = 26)		*p* value
	Mean + SD	Median (IQR)	Mean + SD	Median (IQR)	
Cortisol	116.8 ± 67.2	109.7 (68.4-157.1)	108.8 ± 59.2	104.3 (65.9-143.1)	0.582
Cortisone	106.8 ± 56.1	95.3 (67.0-145.6)	113.6 ± 66.3	97.2 (66.1-152.5)	0.975
Tetrahydrocortisol	1005.7 ± 688.8	878.1 (495.9-1390.8)	1072.2 ± 775.9	769.6 (532.5-1569.4)	0.865
5α-Tetrahydrocortisol	1290.3 ± 810.2	1099.1 (620.2-1707.7)	1551.1 ± 1029.5	1354.7 (852.2-1928.3)	0.331
Tetrahydrocortisone	2526.8 ± 1549.7	2193.4 (1487.2-3389.8)	3158.6 ± 1941.7	2438.8 (1864.9-4380.4)	0.173
Androsterone	1390.1 ± 1359.7	984.3 (361.2-1970.0)	1175.9 ± 870.0	1063.6 (498.1-1540.1)	0.936
Etiocholanolone	767.9 ± 738.5	528.9 (241.3-1089.2)	567.9 ± 417.7	439.1 (328.8-696.8)	0.426
5α-Dihydrotestosterone	14.2 ± 11.5	11.8 (4.5-11.3)	18.2 ± 15.6	12.8 (8.5-19.0)	0.406
Testosterone	20.9 ± 17.0	15.8 (8.7-30.2)	23.6 ± 17.5	20.7 (9.1-38.0)	0.459
Androstenetriol	239.0 ± 255.9	134.5 (44.9-363.8)	184.6 ± 170.3	113.7 (52.0-285.8)	0.625
16α-Hydroxy-DHA	343.8 ± 356.7	228.5 (80.4-496.3)	262.8 ± 259.8	143.5 (65.2-329.9)	0.354
11ß-Hydroxyandrosterone	897.8 ± 657.9	757.2 (419.7-1249.7)	906.4 ± 615.7	810.9 (510.5-1034.4)	0.769
11ß-Hydroxyetiocholanolone	265.2 ± 230.3	201.4 (90.1-377.5)	281.7 ± 335.7	131.4 (79.6-278.5)	0.500
α-Cortol	274.2 ± 206.0	235.2 (116.7-376.2)	284.9 ± 164.3	242.6 (147.7-449.3)	0.395
ß-Cortol	383.3 ± 224.5	331.3 (211.0-504.8)	437.4 ± 321.2	363.6 (183.8-670.0)	0.889
α-Cortolone	1336.0 ± 763.4	1174.3 (765.2-1705.7)	1449.6 ± 781.1	1220.0 (914.5-1936.8)	0.472
ß-Cortolone	415.5 ± 238.7	359.8 (252.0-532.0)	459.2 ± 257.2	417.2 (240.4-573.9)	0.456
